# Eco-phenotypic characterization of *Candida palmioleophila* from poultry litter and marine environments: implications for One Health

**DOI:** 10.1007/s00203-026-05260-3

**Published:** 2026-10-10

**Authors:** Dario Corrêa-Junior, Igor Avellar-Moura, Marcos de Abreu Almeida, Elisa Gonçalves Medeiros, Alessandro F. Valdez, Rodrigo Almeida-Paes, Leonardo Nimrichter, Cláudio Ernesto Taveira Parente, Allan Jefferson Guimarães, Susana Frases

**Affiliations:** 1 Laboratório de Biofísica de Fungos, Instituto de Biofísica Carlos Chagas Filho, Universidade Federal do Rio de Janeiro, Rio de Janeiro, Brazil; 2 Departamento de Microbiologia e Parasitologia, Instituto Biomédico, Universidade Federal Fluminense, Niterói, Brazil; 3 Departamento de Microbiologia Geral, Instituto de Microbiologia Paulo de Goés, Universidade Federal do Rio de Janeiro, Rio de Janeiro, 21941-902 RJ Brazil; 4 Laboratório de Micologia, Instituto Nacional de Infectologia Evandro Chagas, Fundação Oswaldo Cruz, Rio de Janeiro, Brazil; 5 Rede Micologia, FAPERJ, Rio de Janeiro, Brazil; 6 Laboratório de Estudos Ambientais Olaf Malm, Instituto de Biofísica Carlos Chagas Filho, Universidade Federal do Rio de Janeiro, Rio de Janeiro, Brazil

**Keywords:** *Candida palmioleophila*, Antifungal susceptibility, One Health, Biofilm, Poultry litter, Marine environment

## Abstract

*Candida palmioleophila* is an emerging opportunistic yeast frequently misidentified within the *Meyerozyma guilliermondii* complex, potentially obscuring its clinical and ecological relevance. Although its clinical significance is increasingly recognized, the phenotypic characteristics of environmental isolates remain poorly explored. This study evaluated the eco-phenotypic profiles of 14 *C. palmioleophila* isolates recovered from two anthropogenically influenced environmental sources: poultry litter (*n* = 6) and urbanized coastal environments (*n* = 8). Antifungal susceptibility testing revealed high fluconazole MICs among all evaluated isolates (32 to > 128 µg/mL), while amphotericin B, caspofungin, and anidulafungin MICs ranged from 1 to 2 µg/mL, 0.25 to 1 µg/mL, and 0.06 to 0.5 µg/mL, respectively. No detectable association was observed between variation in antifungal MICs and biofilm biomass under the tested conditions. The isolates exhibited broad physiological tolerance, including growth at temperatures up to 40 °C, tolerance to high NaCl concentrations and cell-wall stress, as well as variation in cell-wall chitin content and intracellular lipid-body accumulation. Virulence-associated phenotypes included biofilm formation, high cell-surface hydrophobicity in most isolates, widespread esterase activity, variable phospholipase activity, and limited protease activity. Together, these findings expand current knowledge of the environmental phenotypic repertoire of *C. palmioleophila* and support broader environmental, clinical, and genomic surveillance to clarify its ecology, population structure, and potential transmission pathways.

## Introduction

*Candida palmioleophila* is an emerging non-albicans *Candida* (NAC) species increasingly recognized both as an environmental saprotroph and as an opportunistic human pathogen. Historically, this species has posed a taxonomic challenge; it was frequently misidentified as *Meyerozyma guilliermondii*, *C. famata* (*Debaryomyces hansenii*), or even *Torulopsis candida* due to overlapping biochemical profiles in commercial systems such as Vitek2 and API 20 C (Sugita et al. 1999; Mota et al. 2012). Recent advances in proteomic (MALDI-TOF MS) and molecular methods (ITS and 26 S rDNA sequencing) have substantially improved species identification. Previous studies have reported characteristic turquoise-to-rose colony morphology on CHROMagar and the ability of *C. palmioleophila* to grow at temperatures up to 40 °C, features that may assist its differentiation from closely related species (Jensen and Arendrup 2011; Kothalawala et al. 2019).

The clinical importance of *C. palmioleophila* is increasingly recognized through reports of invasive infections worldwide. Several cases of candidemia caused by this species have been reported in pediatric patients in Iran (Aboutalebian et al. 2023). Furthermore, severe systemic infections in China and Italy highlight its potential to cause severe invasive disease (Kothalawala et al. 2019; Wu et al. 2023). A particular concern is the occurrence of severe clinical manifestations, including endogenous endophthalmitis, together with reports of elevated fluconazole MICs among clinical isolates (Datta et al. 2015; Wu et al. 2023). Genomic studies have begun to identify genetic features potentially associated with antifungal susceptibility and phenotypic variation in this species (Wu et al. 2023; Lavergne et al. 2023).

Beyond the clinical setting, *C. palmioleophila* has been reported across diverse environmental sources. The species has been detected in diverse environmental contexts, including raw sewage in Brazil during SARS-CoV-2 monitoring programs, and has also been reported in marine wildlife, including the green sea turtle (*Chelonia mydas*) (Wang et al. 2021; Costa et al. 2023; Oliveira et al. 2025). Its recent detection in commercially relevant fish from southeastern Brazil further expands the range of environmental and animal-associated sources from which this species has been reported (Oliveira et al. 2025). Collectively, these reports demonstrate that *C. palmioleophila* can occur across diverse environmental and animal-associated settings. However, the phenotypic characteristics that may contribute to its occurrence across such environments remain insufficiently characterized.

In *Candida* spp., several phenotypes have been associated with pathogenic potential, including surface adhesion, biofilm formation, thermotolerance, and the production of hydrolytic enzymes such as proteases and phospholipases (Mroczyńska and Brillowska-Dabrowska 2021). However, these virulence-associated phenotypes remain comparatively understudied in environmental *C. palmioleophila* isolates, and their distribution across isolates from different environmental sources is poorly understood. In addition, previous studies have demonstrated diverse metabolic activities in *C. palmioleophila*, including azo-dye decolorization, tetracycline degradation, and production of enzymes such as L-asparaginase (Jafari et al. 2013; Sisay et al. 2025; Li et al. 2025). The species has also been reported to tolerate high salt concentrations, consistent with its original isolation in medium containing 12.5% NaCl (~ 2.14 M) (Li et al. 2025). This characteristic provides a rationale for investigating stress tolerance in environmental isolates from distinct ecological sources.

From a One Health perspective, the occurrence of *C. palmioleophila* in both environmental and clinical contexts warrants further investigation. Therefore, this study aimed to characterize the eco-phenotypic profiles of environmental *C. palmioleophila* isolates recovered from poultry litter and urbanized coastal environments. Specifically, we evaluated antifungal susceptibility, thermotolerance, stress responses, biofilm formation, cell-surface properties, hydrolytic enzyme activity, and selected structural and metabolic phenotypes. By comparing isolates recovered from two environmental sources, we sought to expand the current understanding of the environmental phenotypic repertoire of *C. palmioleophila* and discuss its potential relevance within a One Health framework.

## Materials and methods

### Environmental samples

A total of 14 environmental isolates of *Candida palmioleophila* were analyzed in this study, comprising six isolates recovered from poultry litter (CP1, CP2, CP3, CP4, CP6, and CP7) and eight isolates recovered from marine environments (I4, J13, J19, J20, J27, J30, J36, and J37) in Rio de Janeiro, Brazil.

Poultry-litter isolates (*n* = 6; CP1, CP2, CP3, CP4, CP6, and CP7) were recovered from two poultry farms, designated BF1 (22°08′57.5″S, 42°52′54.5″W) and BF2 (22°09′38.5″S, 42°56′01.0″W), located in Rio de Janeiro State, Brazil. Isolates CP6 and CP7 were recovered from distinct sampling locations within farm BF2. Poultry litter represents a complex organic matrix characterized by high nutrient content and fluctuating physicochemical conditions. Marine isolates were recovered from seawater from the beaches of Icaraí (22°54′17.0″S, 43°06′59.9″W) and Jurujuba (22°55′42.4″S, 43°06′57.2″W). These sampling sites are located in urbanized coastal areas; however, chemical contaminants were not quantified as part of the present study.

All isolates were subjected to species-level identification because of the close phylogenetic relationship between *Candida palmioleophila* and members of the *Meyerozyma guilliermondii* complex. Initial identification was performed using matrix-assisted laser desorption/ionization time-of-flight mass spectrometry (MALDI-TOF MS) and confirmed by DNA sequencing of the internal transcribed spacer (ITS) region and the D1/D2 domain of the 26 S rRNA gene. Consensus ITS and D1/D2 sequences were compared with nucleotide sequences deposited in the NCBI GenBank database using BLASTn. Species-level identification was assigned based on the best-matching sequences showing ≥ 99% nucleotide identity with *C. palmioleophila*. Molecular sequencing was performed for taxonomic confirmation rather than for population-genetic or strain-level comparison; therefore, genetic relatedness among the environmental isolates was not assessed in the present study.

### Growth kinetics and thermotolerance assay

To characterize growth kinetics and thermotolerance among *C. palmioleophila* isolates from the two environmental sources, fungal growth was evaluated at 25 °C, 37 °C, and 40 °C. Briefly, 200 µL of Sabouraud (SAB) broth was inoculated into 96-well microplates with a standardized yeast suspension. Cell growth was monitored by measuring optical density (OD) at 530 nm using a microplate reader at four time points: 0, 24, 48, and 72 h. All experiments were performed using three independent biological replicates, each analyzed in technical triplicate.

### Antifungal susceptibility test

In vitro antifungal susceptibility assays were performed using the broth microdilution method according to the guidelines of the Clinical and Laboratory Standards Institute (CLSI), document M27-A4 (2015). The antifungal agents evaluated included fluconazole, amphotericin B, caspofungin, and anidulafungin. These antifungal agents were selected to represent three major antifungal classes relevant to the treatment of *Candida* infections: azoles, polyenes, and echinocandins. Stock solutions were prepared in dimethyl sulfoxide (DMSO) and diluted in the test medium to obtain the final working concentrations. The yeast inocula were prepared from 24 to 48-hour-old cultures grown on Sabouraud Dextrose Agar (SDA). Yeast cells were harvested, suspended in RPMI 1640 medium (Sigma-Aldrich) buffered with MOPS (Sigma-Aldrich) at pH 7.0, and adjusted to a final concentration of approximately 0.5 × 10³ to 2.5 × 10³ cells/mL. The assays were conducted in sterile 96-well microtiter plates. Plates were incubated at 35 °C for 24–48 h. The Minimal Inhibitory Concentrations (MIC) were determined visually as the lowest concentration of the drug capable of inhibiting significant fungal growth compared to the drug-free control. The reference strains *Candida krusei* (ATCC 6258) and *Candida parapsilosis* (ATCC 22019) were included as quality controls in all assays. MICs were determined using the CLSI broth microdilution methodology. MIC values for the environmental *C. palmioleophila* isolates were reported quantitatively because categorical interpretation requires species- and antifungal-specific clinical breakpoints or epidemiological cutoff values.

### Plate assays for extracellular hydrolytic enzymes

Hydrolytic enzyme activities were evaluated as virulence-associated phenotypes using specific agar plate assays. Phospholipase activity was determined using egg yolk agar (SDA supplemented with 1 M NaCl, 5 mM CaCl_2_, and 8% sterile egg yolk emulsion, pH 7.0). Esterase activity was assessed using Tween 80 agar (1% peptone, 0.5% yeast extract, 0.01% CaCl_2_, 0.1% Tween 80, and 1.5% agar, pH 7.0). Protease activity was evaluated following standard protocols for protein degradation. Briefly, 5 µL aliquots of a 10^6^ cells/mL suspension were deposited onto the media surface and incubated at 37°C for 7 days. The enzymatic activity was quantified by the Pz value (a/b), where ‘a’ is the colony diameter and ‘b’ is the total diameter (colony + degradation zone). Pz values were classified as: excellent producers (Pz < 0.399), good producers (0.400 < Pz < 0.699), poor producers (0.700 < Pz < 0.999), and non-producers (Pz = 1.0). *Candida krusei* (ATCC6258) and *Candida parapsilosis* (ATCC22019) were used as controls of each culture medium (Price et al. 1982).

### Biofilm formation on polystyrene

A 96-well plate method was used to evaluate biofilm development. Candida palmioleophila isolates were cultured on Sabouraud Dextrose Agar (SDA) at 37 °C for 24 h. The yeast cells were suspended in phosphate-buffered saline (PBS), and the concentration was adjusted to 1 × 10⁶ cells/mL using a hemocytometer. Subsequently, 100 µL of the standardized suspension was inoculated into wells of a sterile, flat-bottom 96-well microtiter plate and incubated at 37 °C for 2 h to allow pre-adhesion. Wells were then gently washed with PBS to remove non-adherent cells, and 200 µL of RPMI 1640 medium (with L-glutamine, without bicarbonate) buffered to pH 7.0 with MOPS was added. Plates were incubated at 25 °C and 37 °C for 72 h to allow biofilm maturation. The experiment was conducted with three biological replicates (*n* = 3), and each measurement was performed in triplicate. Each assay included a negative control consisting of sterile RPMI medium without fungal inoculation (Rehman et al. 2025).

### Biomass quantification

Biofilms were fixed with 200 µL of 99% methanol (Sigma-Aldrich, St. Louis, MO, USA) for 15 min. Supernatants were discarded, and microplates were air-dried for 5 min. Then, 200 µL of a 0.5% crystal violet solution (diluted in PBS; Sigma-Aldrich, St. Louis, MO, USA) were added to each well and incubated for 20 min at 25 °C. The dye was removed by washing with distilled water, and the biomass was decolorized with 200 µL of 33% acetic acid (v/v) for 5 min. Aliquots of 100 µL of the decolorized solution were transferred to a new 96-well plate (Jet Biofil, Huangpu, Guangzhou, China), and absorbance was measured at 590 nm using a microplate reader (SpectraMax M3; Molecular Devices, San José, CA, USA) (Marcos-Zambrano et al. 2014).

### Extracellular matrix-associated staining

To evaluate extracellular matrix-associated staining, biofilms were stained with 200 µL of 0.1% safranin in PBS (Sigma-Aldrich, St. Louis, MO, USA) for 5 min at room temperature. Wells were then washed with PBS until the supernatants were clear. Decolorization was performed with 200 µL of 30% acetic acid (v/v) for 5 min. Finally, 100 µL of the resulting supernatants were transferred to a new 96-well plate (Jet Biofil, Huangpu, Guangzhou, China), and absorbance was measured at 530 nm using the SpectraMax M3 microplate reader, as previously described (Mello et al. 2018).

### Assessment of cell surface hydrophobicity

Cell surface hydrophobicity (CSH) was measured using the two-phase water–octane assay. A 1.2 mL volume of a 1 × 10⁶ yeast cells/mL suspension was washed with phosphate-buffered saline (PBS) and vigorously shaken with 0.3 mL of anhydrous octane (Sigma–Aldrich, St. Louis, MO, USA). The mixture was then left undisturbed for 15 min at room temperature. The absorbance at 600 nm (ABS₆₀₀) of fungal cells in PBS without octane was used as a negative control. The percentage of fungal cell exclusion from the aqueous phase (i.e., the % change in ABS₆₀₀), representing relative cellular hydrophobicity, was calculated using the formula: ([ABS₆₀₀ from control – ABS₆₀₀ after octane overlay] / ABS₆₀₀ from control) × 100. As previously described, CSH levels were classified as follows: High: 80%–100% change in ABS₆₀₀, Moderate: 20%–80% and Low: 0%–20% change in ABS₆₀₀ (Hazen et al. 1986; Ramos et al. 2020).

### Assessment of cell surface charge (zeta potential)

The Zeta potential (ζ), particle mobility and shift frequency of samples were calculated in a Zeta potential analyzer (NanoBrook Omni Zeta Potential Analytical Instruments Particle Sizer, Brookhaven Instruments Corp., Holtsville, NY, USA). This is a measurement of charge (in millivolts) defined as the potential gradient that develops across the interface between a boundary liquid in contact with a solid and the mobile diffuse layer in the body of the liquid. The Zeta potential was evaluated using a yeast cell suspension (1.5 mL containing 10⁶ cells/mL) prepared in 1 mM NaCl in ultrapure water at 25 °C and pH 6.0. All measurements were performed in 10 cycles (de Araújo et al. 2019).

### Quantification of chitin in the cell wall

Yeast suspensions were prepared as described above and stained with Uvitex 2B (Polysciences Inc., Warrington, PA, USA) to quantify cell-wall chitin. Briefly, 100 µL of Uvitex 2B (10 µg/mL) was added to 1 mL of the yeast suspension. Samples were incubated at 37 °C for 60 min, washed three times with PBS, and analyzed by flow cytometry (BD Biosciences, San Jose, CA, USA) (Ramos et al. 2020).

### Detection of lipid bodies in the fungal cell

Intracellular lipid-body accumulation was quantified using Nile Red staining. Yeast suspensions (10⁶ cells/mL) were fixed with paraformaldehyde (Sigma-Aldrich, St. Louis, MO, USA) and stained with Nile Red (Sigma-Aldrich, St. Louis, MO, USA) at 5 µg/mL for 30 min at room temperature. Yeast cells treated with PBS alone were used as autofluorescence controls. Subsequently, cells were washed three times with PBS and analyzed by flow cytometry (BD Biosciences, San Jose, CA, USA). A total of 10,000 events were acquired, and the gated population was analyzed for cell size and red fluorescence using a single-parameter histogram. Results were expressed as mean fluorescence intensity (MFI), as previously described (Corrêa-Junior et al. 2023).

### Phenotypic stress tolerance assay

To evaluate phenotypic stress tolerance among *C. palmioleophila* isolates, a spot assay was performed under graded stress conditions. Serially diluted fungal suspensions (10⁶ to 10² cells/mL) were spotted onto Sabouraud Dextrose Agar (SDA) supplemented with sodium chloride (NaCl; 1.0, 1.5, and 2.0 M) as an osmotic stressor; Congo Red (10, 50, and 100 µg/mL) as a cell-wall stressor; sodium dodecyl sulfate (SDS; 0.02%, 0.04%, and 0.06% w/v) as a membrane stressor; or caffeine (10, 20, and 30 mM) as a chemical stressor. Plates were incubated at 25 °C and 37 °C for 72 h, with unsupplemented SDA serving as the growth control (Huang et al. 2024).

### Statistical analysis

All quantitative experiments, including growth kinetics, biofilm biomass, extracellular matrix-associated staining, cell-surface hydrophobicity (CSH), flow-cytometry measurements (chitin content and intracellular lipid-body mean fluorescence intensity [MFI]), and hydrolytic enzyme activities (Pz values), were performed using three independent biological replicates, each analyzed in technical triplicate. Data are presented as mean ± standard deviation (SD).

Statistical analyses were performed in R version 4.3.2 (R Foundation for Statistical Computing, Vienna, Austria). Generalized linear models (GLMs) with a Gamma error distribution and log-link function were fitted using the stats package, whereas estimated marginal means (EMMs) were calculated using the emmeans package. A Gamma distribution was selected because the response variables consisted of continuous, strictly positive, right-skewed measurements. The models were used to explore statistical associations among the measured phenotypic variables.

To explore whether variation in fluconazole MIC was associated with biofilm biomass at 25 °C, an additional GLM was fitted using biofilm biomass as the response variable and fluconazole MIC as a categorical explanatory variable, with phospholipase activity included as a covariate. Fluconazole MIC was treated as a categorical variable according to the four MIC levels observed among the environmental isolates (32, 64, 128, and > 128 µg/mL). Values reported as > 128 µg/mL were retained as a distinct right-censored category and were not assigned an arbitrary numerical value for statistical analysis. A separate Gamma GLM was fitted to explore the association between amphotericin B MIC values and biofilm biomass at 25 °C. A separate Gamma GLM was fitted to evaluate differences in phospholipase activity between the two environmental sources examined (poultry litter and marine environments).

Estimated marginal means (EMMs), pairwise comparisons, and predicted values were obtained using the *emmeans* package. Model estimates were back-transformed from the log scale to the original response scale, and uncertainty was expressed as 95% confidence intervals (95% CI).

For visualization of antifungal susceptibility profiles, minimum inhibitory concentration (MIC) values for amphotericin B, caspofungin, and anidulafungin were log₂-transformed to preserve the geometric dilution intervals used in broth microdilution assays. The transformed data were plotted in a three-dimensional Cartesian space. A maximum coordinate jitter of 0.04 was applied exclusively to the graphical representation of data points to avoid overlap among isolates with identical MIC profiles without altering the original MIC values (Wickham 2016). Statistical analyses and graphical visualization were performed using R and GraphPad Prism (version 9.0). Statistical significance was defined as *p* < 0.05.

## Results

### Thermotolerance and growth dynamics

**Fig. 1:**
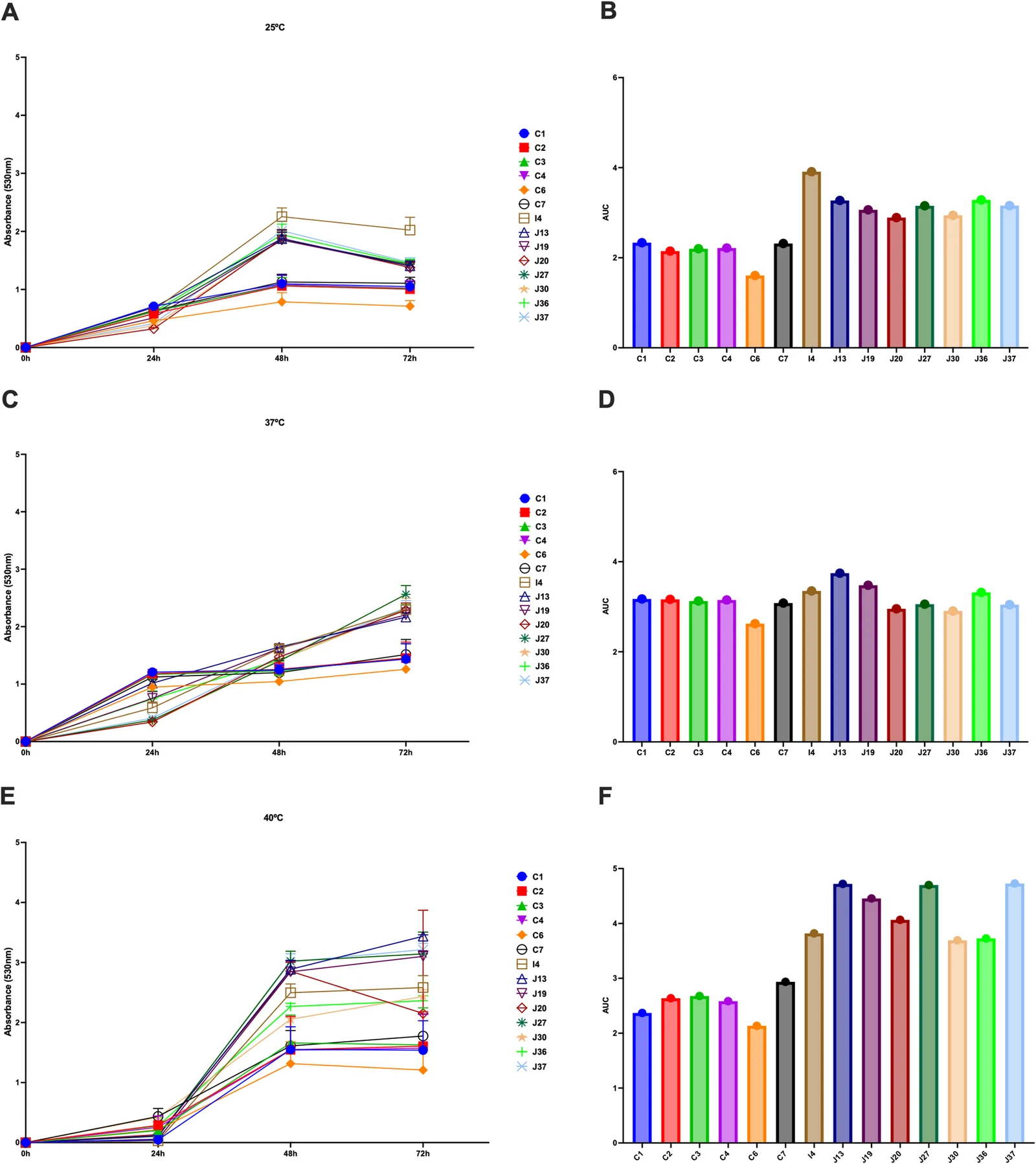
Growth kinetics and area under the curve (AUC) analysis of environmental *Candida palmioleophila* isolates at different temperatures. Growth was monitored by optical density at 530 nm over 72 h. **A**–**B** Growth kinetics and corresponding AUC values at 25 °C. **C**–**D** Growth kinetics and corresponding AUC values at 37 °C. **E**–**F** Growth kinetics and corresponding AUC values at 40 °C. Data represent the mean ± standard deviation of three independent biological experiments, each performed in technical triplicate. For graphical presentation, poultry-litter isolates are designated by the prefix C, whereas marine isolates are designated by I or J

All 14 environmental *C. palmioleophila* isolates exhibited measurable growth across the tested temperature range (25 °C, 37 °C, and 40 °C) (Fig. 1). At 25 °C, the isolates reached a stationary phase between 48 and 72 h (Fig. 1A), with the corresponding Area Under the Curve (AUC) measurements summarizing overall growth during the 72-h incubation period (Fig. 1B). At 37 °C, growth was sustained throughout the incubation period in isolates from both poultry-litter and marine sources (Fig. 1C and D).

At 40 °C, all isolates exhibited a prolonged initial lag phase during the first 24 h, followed by increased growth at 48 and 72 h (Fig. 1E). This growth pattern was observed among isolates from both environmental sources and was reflected in the corresponding AUC measurements (Fig. 1F). Overall, these findings demonstrate that the environmental *C. palmioleophila* isolates evaluated in this study were able to grow at temperatures up to 40 °C, with isolate-dependent variation in growth kinetics across the tested temperatures.

### Antifungal susceptibility profile

All 14 environmental *Candida palmioleophila* isolates exhibited high fluconazole MICs, ranging from 32 to > 128 µg/mL (Table 1). The fluconazole MIC50 and MIC90 were 128 and > 128 µg/mL, respectively. Amphotericin B MICs ranged from 1 to 2 µg/mL, with an MIC50 of 1 µg/mL and an MIC90 of 2 µg/mL. Four isolates (CP1, CP2, CP7, and J27) exhibited an amphotericin B MIC of 2 µg/mL.

Caspofungin MICs ranged from 0.25 to 1 µg/mL, with MIC50 and MIC90 values of 0.5 and 1 µg/mL, respectively. Anidulafungin MICs ranged from 0.06 to 0.5 µg/mL, with an MIC50 of 0.12 µg/mL and an MIC90 of 0.25 µg/mL. Because species-specific categorical interpretive criteria for *C. palmioleophila* were not applied, the antifungal susceptibility results are presented as quantitative MIC distributions rather than as susceptible or resistant categories.

**Table 1:** In vitro antifungal MIC profiles of environmental *Candida palmioleophila* isolates

Isolate	Fluconazole	Amphotericin B	Caspofungin	Anidulafungin
CP1	128	2	0.25	0.06
CP2	64	2	0.5	0.06
CP3	**> 128**	**1**	**0.5**	**0.06**
CP4	**> 128**	**1**	**1**	**0.25**
CP6	**32**	**1**	**1**	**0.25**
CP7	**128**	**2**	**0.5**	**0.06**
I4	**128**	**1**	**0.5**	**0.12**
J13	**> 128**	**1**	**0.5**	**0.12**
J19	**64**	**1**	**1**	**0.25**
J20	**128**	**1**	**1**	**0.5**
J27	**128**	**2**	**0.25**	**0.06**
J30	**128**	**1**	**0.5**	**0.12**
J36	**128**	**1**	**0.5**	**0.12**
J37	**128**	**1**	**0.5**	**0.12**
MIC50	**128**	**1**	**0.5**	**0.12**
MIC90	**> 128**	**2**	**1**	**0.25**
*C. parapsilosis* ATCC 22,019 (QC)	**8**	**2**	**1**	**2**
*C. krusei* ATCC 6258 (QC)	**> 128**	**4**	**1**	**0.12**

Minimum inhibitory concentrations (MICs) are expressed in µg/mL. CP indicates poultry-litter isolates, whereas I and J indicate marine isolates. MIC50 and MIC90 values were calculated from the MIC distributions of the 14 environmental isolates and represent the concentrations encompassing at least 50% and 90% of the isolates, respectively. Quality-control strains were excluded from MIC50 and MIC90 calculations. Fluconazole values above the highest concentration tested (> 128 µg/mL) were retained as right-censored values. *Candida parapsilosis* ATCC 22,019 and *Candida krusei* ATCC 6258 were included as quality-control strains. MICs were determined by broth microdilution according to CLSI methodology after 24–48 h of incubation at 35 °C

### Three-dimensional antifungal MIC mapping

**Fig. 2:**
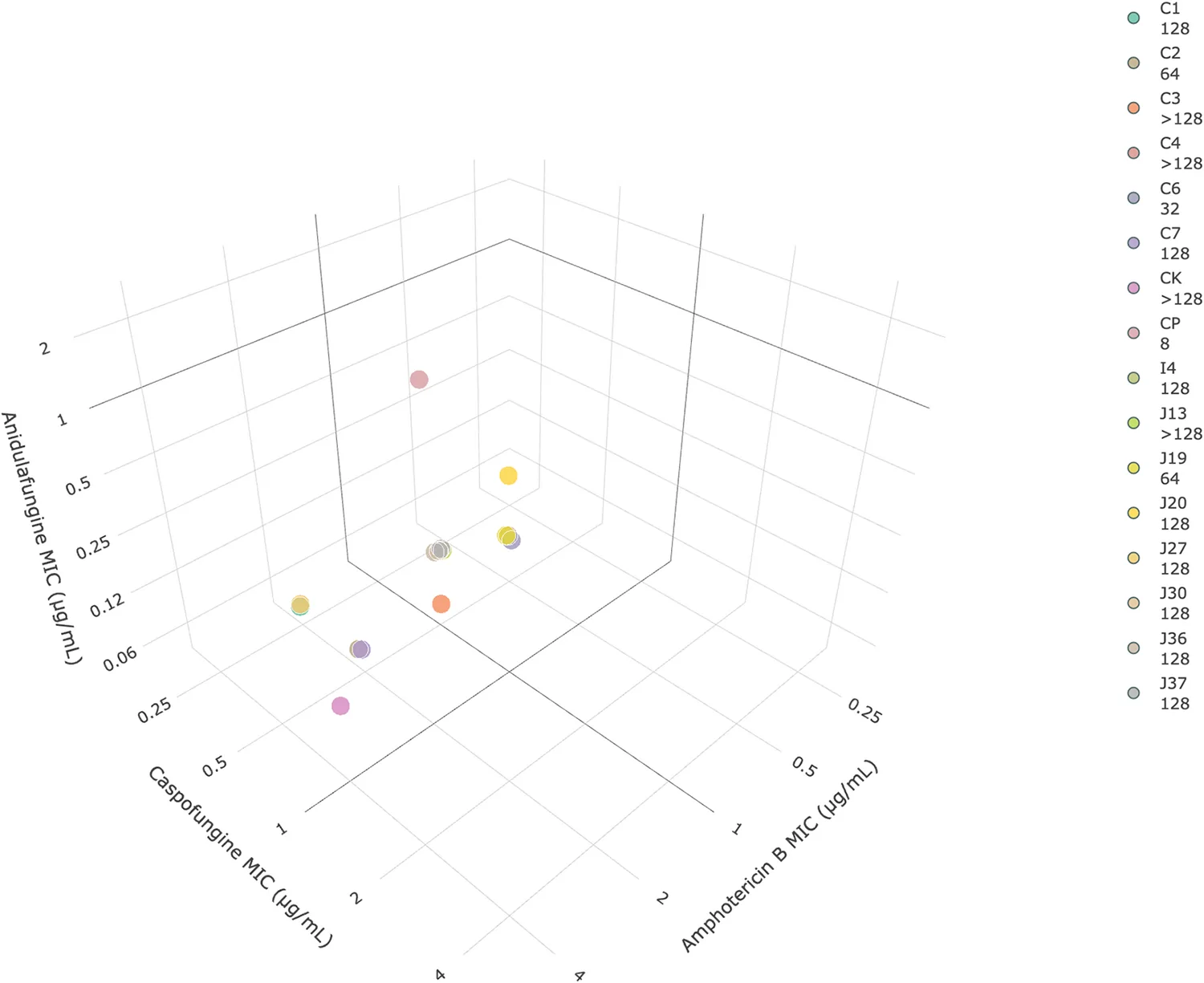
Three-dimensional distribution of non-azole antifungal MIC profiles among environmental *Candida palmioleophila* isolates. Minimum inhibitory concentrations (MICs) for amphotericin B, caspofungin, and anidulafungin were log₂-transformed and represented in three-dimensional Cartesian space. For graphical presentation, poultry-litter isolates are designated by the prefix C, whereas marine isolates are designated by I or J. Coordinate jittering was applied exclusively for graphical visualization to distinguish overlapping isolates with identical MIC profiles and did not alter the original MIC values

To visualize the distribution of non-azole antifungal MIC profiles among the environmental isolates, amphotericin B, caspofungin, and anidulafungin MIC values were represented in a three-dimensional log₂-transformed space (Fig. 2). Most isolates clustered within a relatively narrow range of caspofungin (0.25–1 µg/mL) and anidulafungin (0.06–0.5 µg/mL) MICs.

Variation in the three-dimensional distribution was primarily associated with amphotericin B MIC values. Four isolates (CP1, CP2, CP7, and J27) exhibited amphotericin B MICs of 2 µg/mL, whereas the remaining environmental isolates exhibited MICs of 1 µg/mL. Coordinate jittering was applied exclusively for visualization to distinguish isolates with identical MIC profiles and did not alter the original MIC values (Fig. 2).

### Hydrolytic enzyme activity

Environmental *C. palmioleophila* isolates exhibited heterogeneous hydrolytic enzyme activity. Esterase activity was the most frequently detected phenotype, with 13 of the 14 isolates (92.9%) classified as excellent producers (Pz < 0.399), whereas CP3 was classified as a good producer.

Phospholipase activity varied among isolates. CP3, CP4, and CP7 were classified as non-producers (Pz = 1.0), whereas J19 exhibited excellent phospholipase activity and J20 and CP2 were classified as good producers. Protease activity was uncommon among the evaluated isolates, with most isolates classified as non-producers (Pz = 1.0). CP4 was the exception, exhibiting a Pz value of 0.3 and being classified as an excellent producer.

Overall, esterase activity was widespread among the evaluated isolates, whereas phospholipase activity was variable and protease activity was uncommon.

### Biofilm formation and extracellular matrix-associated staining

**Fig. 3:**
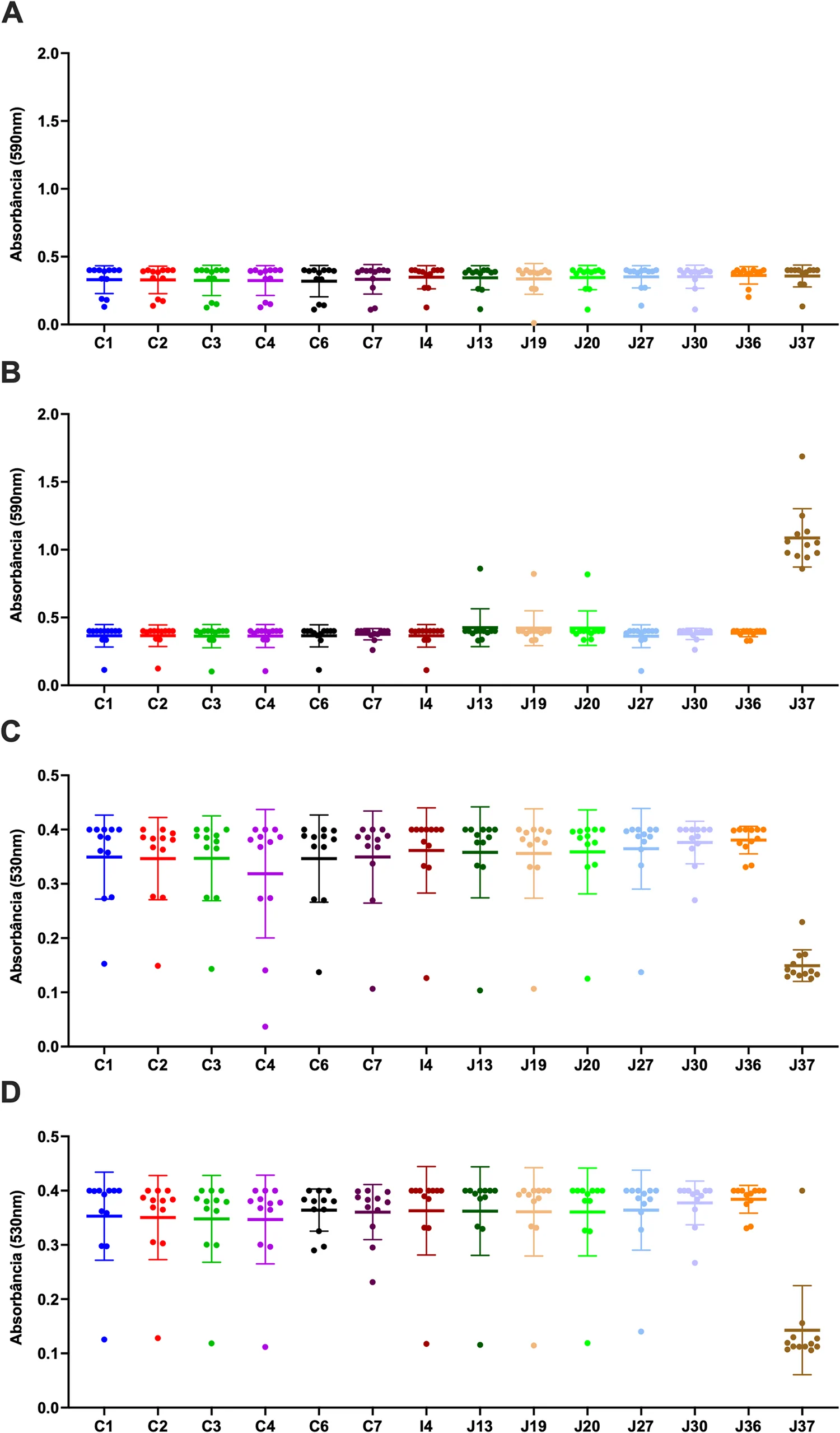
Biofilm biomass and extracellular matrix-associated staining of environmental *Candida palmioleophila* isolates at 25 °C and 37 °C. **A**–**B** Biofilm biomass measured by crystal violet staining at 590 nm after incubation at 25 °C and 37 °C, respectively. **C**–**D** Extracellular matrix-associated staining measured by safranin staining at 530 nm at 25 °C and 37 °C, respectively. Data represent the mean ± standard deviation of three independent biological experiments, each performed in technical triplicate. For graphical presentation, poultry-litter isolates are designated by the prefix C, whereas marine isolates are designated by I or J

All environmental *C. palmioleophila* isolates formed measurable biofilm biomass on polystyrene at both 25 °C and 37 °C (Fig. 3). At 25 °C, biofilm biomass values were relatively similar among most isolates from the two environmental sources (Fig. 3A). At 37 °C, greater inter-isolate variability was observed, with the marine isolate J37 exhibiting the highest biofilm biomass among the evaluated isolates (Fig. 3B).

Extracellular matrix-associated staining with safranin was detected among isolates at both temperatures (Fig. 3C and D). J37, despite exhibiting comparatively high biofilm biomass at 37 °C, showed lower safranin staining relative to its biomass measurement. Overall, biofilm biomass and extracellular matrix-associated staining varied among isolates and between the tested temperatures.

### Cell surface hydrophobicity (CSH)

**Fig. 4:**
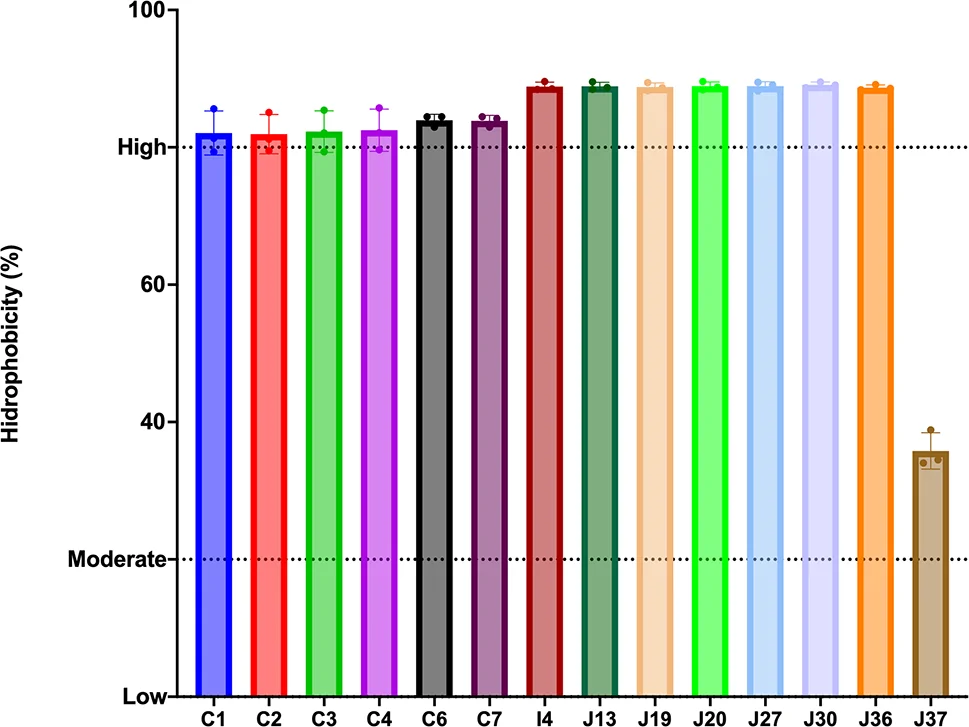
Cell-surface hydrophobicity (CSH) of environmental *Candida palmioleophila* isolates. Relative hydrophobicity was determined by the percentage of cell exclusion from the aqueous phase in a water–octane assay. CSH levels were classified as high (80%–100%), moderate (20%–80%), or low (0%–20%). Data are presented as the mean ± standard deviation of three independent experiments. For graphical presentation, poultry-litter isolates are designated by the prefix C, whereas marine isolates are designated by I or J

Cell-surface hydrophobicity varied among the environmental *C. palmioleophila* isolates (Fig. 4). Most isolates from both poultry-litter and marine sources exhibited CSH values above 80% and were therefore classified as highly hydrophobic according to the criteria used in this study. In contrast, the marine isolate J37 exhibited a CSH value of approximately 35%, corresponding to moderate hydrophobicity. Overall, high CSH was the predominant phenotype among the evaluated isolates, with J37 representing a distinct lower-hydrophobicity phenotype.

### Assessment of cell surface charge (Zeta Potential)

**Fig. 5:**
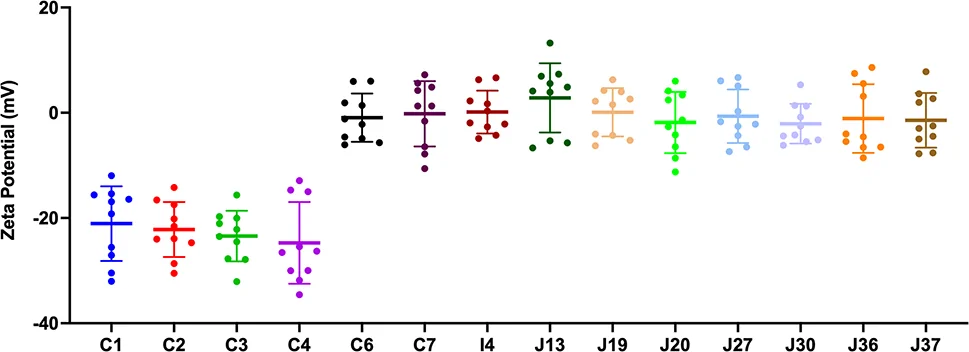
Zeta-potential measurements of environmental *Candida palmioleophila* isolates. Cell-surface charge (mV) was measured for 14 environmental isolates recovered from poultry litter and marine environments. Measurements were performed in 1 mM NaCl at 25 °C and pH 6.0. Individual data points represent measurements obtained across 10 cycles per isolate, and horizontal bars indicate the mean ± standard deviation. For graphical presentation, poultry-litter isolates are designated by the prefix C, whereas marine isolates are designated by I or J

Zeta-potential measurements revealed substantial variation in cell-surface charge among the environmental *C. palmioleophila* isolates (Fig. 5). Poultry-litter isolates CP1 (− 21.07 ± 7.09 mV), CP2 (− 22.18 ± 5.31 mV), CP3 (− 23.43 ± 4.92 mV), and CP4 (− 24.73 ± 7.85 mV) exhibited the most negative Zeta-potential values. In contrast, the remaining isolates, including poultry-litter isolates CP6 and CP7 and the marine isolates, exhibited values closer to neutrality. Among these isolates, values ranged from − 2.15 ± 3.92 mV for J30 to + 2.93 ± 6.65 mV for J13. These findings demonstrate inter-isolate variation in the electrostatic properties of the cell surface, with CP1–CP4 exhibiting a more negative surface charge than the remaining isolates.

### Chitin content and intracellular lipid-body accumulation

**Fig. 6:**
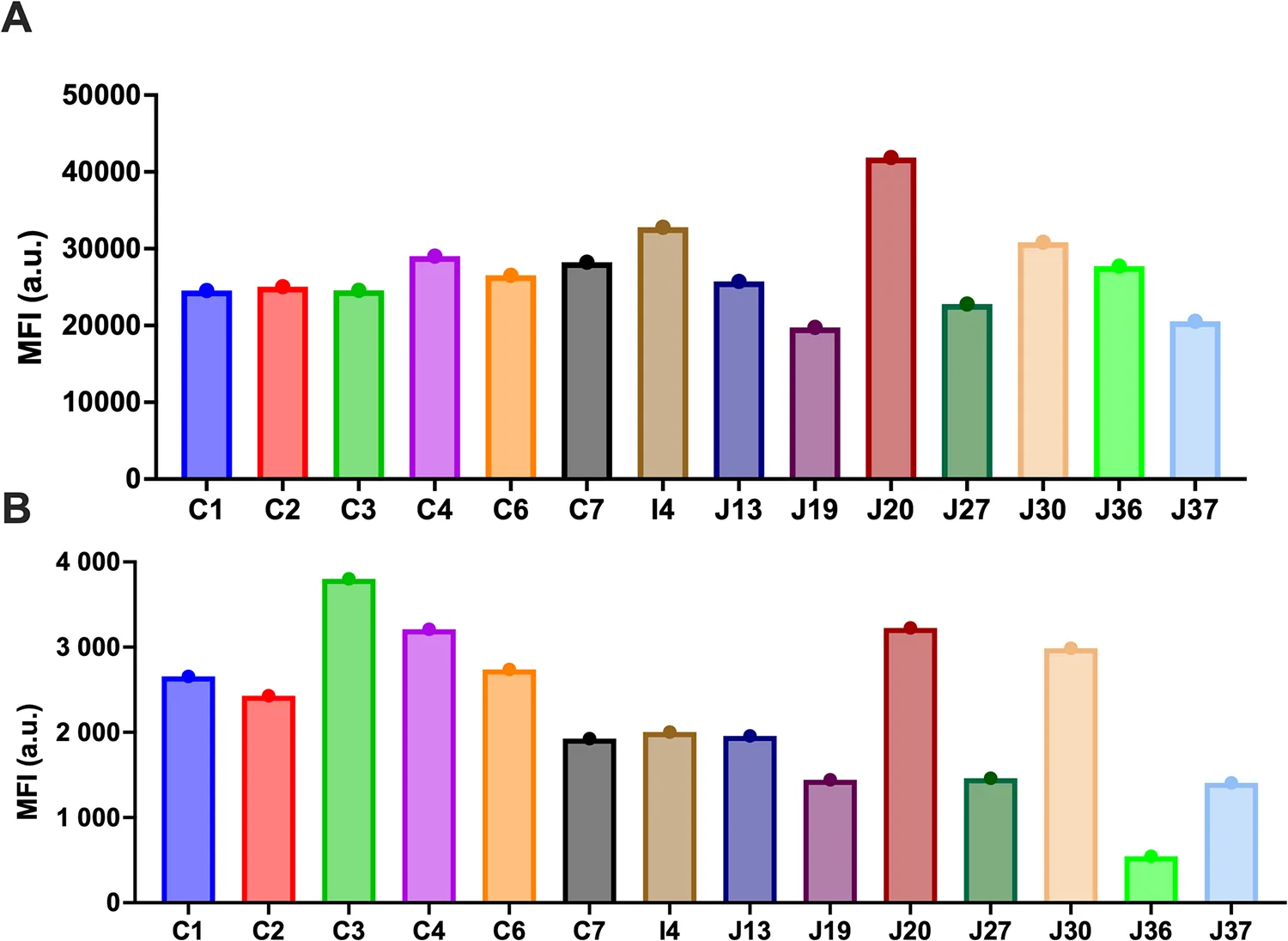
Structural and metabolic markers of environmental *Candida palmioleophila* isolates. **A** Cell-wall chitin content quantified by Uvitex 2B fluorescence and expressed as mean fluorescence intensity (MFI). **B** Intracellular lipid-body accumulation quantified by Nile Red staining and expressed as MFI. Data are expressed as mean fluorescence intensity (a.u.) ± standard deviation from independent experiments. For graphical presentation, poultry-litter isolates are designated by the prefix C, whereas marine isolates are designated by I or J

Cell-wall chitin content varied among the environmental *C. palmioleophila* isolates (Fig. 6). Uvitex 2B fluorescence values were predominantly distributed between approximately 20,000 and 30,000 arbitrary units (a.u.), whereas the marine isolates J20 and J30 exhibited comparatively higher values, with J20 exceeding 40,000 a.u.

Intracellular lipid-body accumulation, quantified by Nile Red staining, also varied among isolates. Higher fluorescence values (> 3,000 a.u.) were observed in poultry-litter isolates CP3 and CP4 and in marine isolates J20 and J30. Overall, both cell-wall chitin content and intracellular lipid-body accumulation showed inter-isolate variability.

### Gradient stress tolerance profiling

**Fig. 7:**
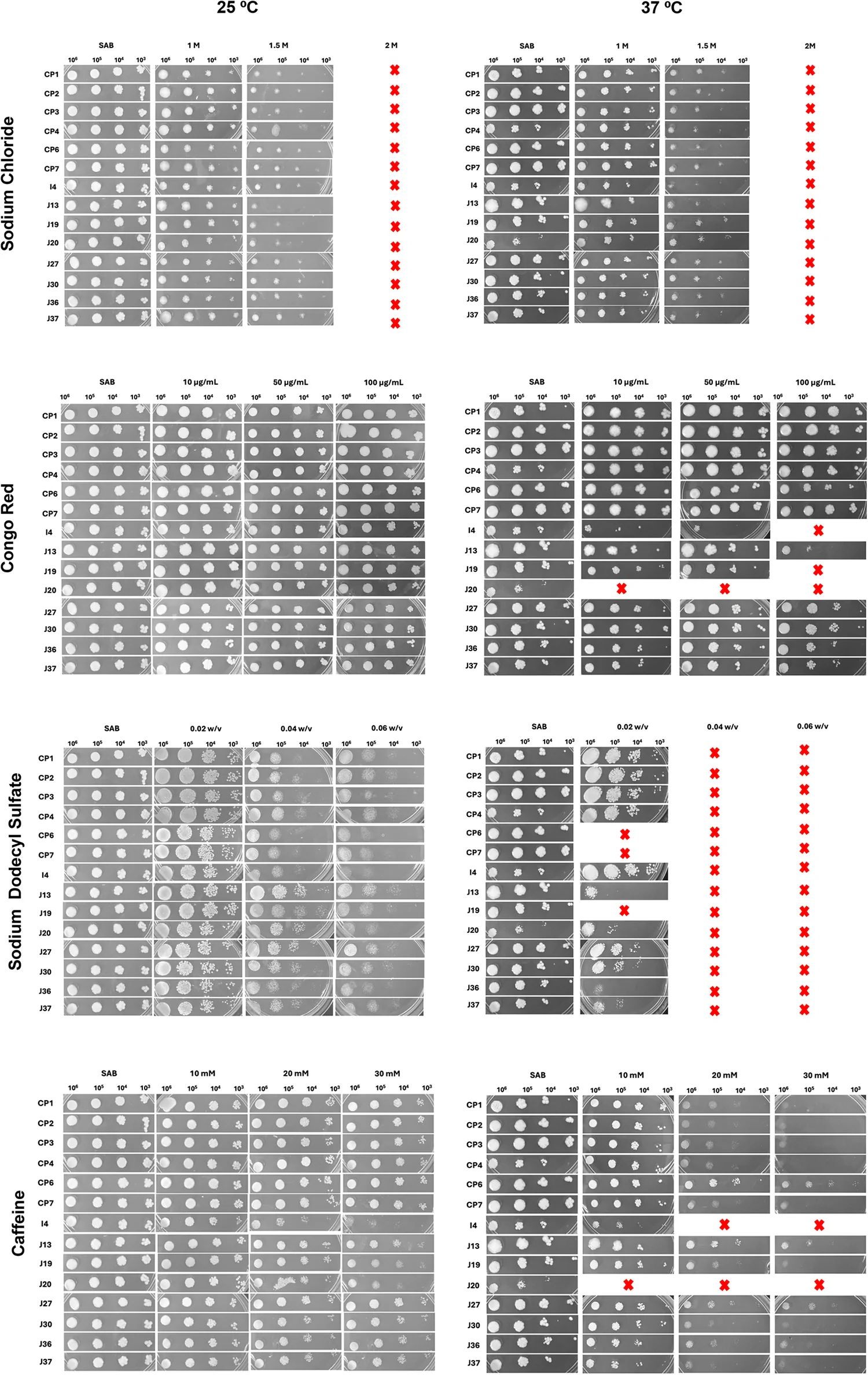
Comparative stress tolerance of environmental *Candida palmioleophila* isolates under graded stress conditions at 25 °C and 37 °C. Serial dilutions (10⁶ to 10² cells/mL) of the 14 environmental isolates were grown on Sabouraud Dextrose Agar (SDA) supplemented with NaCl (1.0, 1.5, and 2.0 M), Congo Red (10, 50, and 100 µg/mL), SDS (0.02%, 0.04%, and 0.06% w/v), or caffeine (10, 20, and 30 mM). Unsupplemented SDA served as the growth control. Poultry-litter isolates are designated by the prefix CP, whereas marine isolates are designated by I or J. Red “X” symbols indicate absence of detectable growth under the corresponding experimental condition

The environmental *C. palmioleophila* isolates exhibited growth under multiple stress conditions, with responses varying according to stressor concentration (Fig. 7). Under osmotic stress, all isolates maintained growth in the presence of 1.0 and 1.5 M NaCl. At 2.0 M NaCl, a reduction in colony growth was observed, although growth remained detectable across the tested isolates. Growth was also maintained in the presence of Congo Red at concentrations up to 100 µg/mL. In contrast, increasing SDS concentrations resulted in greater growth inhibition: growth was maintained at 0.02% SDS, markedly reduced at 0.04%, and inhibited at 0.06% under the tested conditions. Caffeine exposure resulted in concentration-dependent growth inhibition, with stronger inhibition observed at 20 and 30 mM, particularly for isolate CP6.

**Table 2:** Comparative phenotypic characteristics of environmental *Candida palmioleophila* isolates recovered from poultry litter and marine environments

Characteristic	Poultry litter isolates (*n* = 6)	Marine isolates (*n* = 8)	Main finding
Fluconazole MIC	32 to > 128 µg/mL	64 to > 128 µg/mL	High MICs observed in both environmental groups
Amphotericin B MIC	1–2 µg/mL	1–2 µg/mL	Similar MIC ranges between groups
Caspofungin MIC	0.25–1 µg/mL	0.25–1 µg/mL	Similar MIC ranges between groups
Anidulafungin MIC	0.06–0.25 µg/mL	0.06–0.5 µg/mL	Quantitative MIC distributions in both groups
NaCl stress	Growth maintained up to 2.0 M	Growth maintained up to 2.0 M	Growth maintained under the tested NaCl conditions
Congo Red stress	Growth maintained up to 100 µg/mL	Growth maintained up to 100 µg/mL	Growth maintained under cell-wall stress
SDS stress	Concentration-dependent inhibition	Concentration-dependent inhibition	Marked reduction at 0.04%; inhibition at 0.06%
Caffeine stress	Concentration-dependent inhibition	Concentration-dependent inhibition	Stronger inhibition at 20–30 mM
Cell-surface hydrophobicity	Predominantly high	Predominantly high	J37 exhibited moderate CSH
Esterase activity	Predominantly high	Predominantly high	Esterase activity was widespread
Phospholipase activity	Variable	Variable	Inter-isolate variation was observed
Protease activity	Uncommon	Uncommon	Most isolates were non-producers
Growth at 37 °C	Maintained	Maintained	All isolates grew at 37 °C
Growth at 40 °C	Maintained	Maintained	All isolates showed growth at 40 °C
Cell-wall chitin content	Variable	Variable	Inter-isolate variation was observed
Intracellular lipid bodies	Variable	Variable	Higher values occurred in selected isolates from both sources

Summary of antifungal MIC distributions, stress-tolerance profiles, cell-surface hydrophobicity (CSH), hydrolytic enzyme activity, thermotolerance, cell-wall chitin content, and intracellular lipid-body accumulation among poultry-litter (*n* = 6) and marine (*n* = 8) isolates. The table summarizes experimentally measured phenotypes and does not imply environmental selection, reservoir status, or direct pathogenic potential

### Statistical associations among measured phenotypic traits

Fluconazole MIC category was not significantly associated with biofilm biomass at 25 °C under the tested conditions. Estimated marginal means showed overlapping confidence intervals among the evaluated MIC categories, indicating no detectable association between variation in fluconazole MIC and biofilm biomass.

Similarly, no detectable association was observed between amphotericin B MIC values (1–2 µg/mL among the environmental isolates) and biofilm biomass at 25 °C. Phospholipase activity did not differ significantly between the two environmental sources examined (poultry litter and marine environments).

These analyses therefore did not identify detectable associations between the evaluated antifungal MIC variables and biofilm biomass under the tested conditions. These findings are restricted to the measured phenotypic outcomes and should not be interpreted as evidence for the presence or absence of an overall biological fitness cost.

## Discussion

*Candida palmioleophila* is an emerging opportunistic yeast that remains challenging to identify because of its phenotypic similarity to members of the *Meyerozyma guilliermondii* complex (Sugita et al. 1999; Mota et al. 2012). In the present study, we characterized 14 environmental isolates recovered from poultry litter and urbanized coastal environments, integrating antifungal susceptibility testing with measurements of thermotolerance, stress responses, biofilm formation, cell-surface properties, hydrolytic enzyme activity, and selected structural and metabolic phenotypes. These data expand the available phenotypic information for environmental isolates of this understudied species.

A notable finding of the present study was the consistently high fluconazole MIC distribution among the 14 environmental isolates, ranging from 32 to > 128 µg/mL. Elevated fluconazole MICs have previously been reported for *C. palmioleophila*, reinforcing the importance of further investigating antifungal susceptibility in this species (Jensen and Arendrup 2011) (Lavergne et al. 2023). Amphotericin B MICs ranged from 1 to 2 µg/mL, whereas caspofungin and anidulafungin MICs ranged from 0.25 to 1 µg/mL and from 0.06 to 0.5 µg/mL, respectively. The occurrence of high fluconazole MICs in isolates from both environmental sources raises questions regarding the factors contributing to the maintenance of this phenotype. However, because environmental antifungal compounds, agrochemicals, and other chemical contaminants were not quantified and the study did not include comparisons with less anthropogenically influenced sites, the present data cannot determine whether the observed MIC profiles resulted from local environmental selection or represent intrinsic or pre-existing characteristics of the isolates (Costa et al. 2023).

The ability of all evaluated isolates to grow at 37 °C and maintain growth at 40 °C demonstrates substantial thermotolerance. This phenotype is consistent with previous observations for *C. palmioleophila* (Jensen and Arendrup 2011) and indicates that environmental isolates of this species can tolerate a relatively broad temperature range. However, growth at these temperatures alone does not demonstrate adaptation to avian or human hosts or the capacity for host transition.

The isolates also maintained growth under high NaCl concentrations and in the presence of Congo Red, demonstrating tolerance to the osmotic and cell-wall stress conditions tested. Salt tolerance has previously been reported in *C. palmioleophila* (Li et al. 2025). In contrast, increasing concentrations of SDS and caffeine resulted in progressive growth inhibition, demonstrating differential responses according to the type and intensity of the stressor. Although cross-protection between environmental stress responses and antifungal susceptibility has been proposed for fungi, the present experiments do not establish mechanistic links between stress tolerance and antifungal MIC variation.

Cell-wall chitin content and intracellular lipid-body accumulation varied among the environmental isolates. Comparatively high chitin fluorescence was observed in some marine isolates, whereas increased lipid-body accumulation occurred in selected isolates from both environmental sources. These cellular components have been associated with fungal stress responses in other contexts; however, their functional contribution to stress tolerance or antifungal susceptibility was not directly evaluated in the present study. Therefore, the observed variation should be interpreted as phenotypic diversity rather than evidence of specific adaptive mechanisms.

Biofilm formation and high cell-surface hydrophobicity (CSH) were observed among most isolates. Both phenotypes have been associated with surface interactions and biofilm development in *Candida* spp. (Mroczyńska and Brillowska-Dabrowska 2021); however, adhesion itself was not independently quantified in the present study. Notably, the marine isolate J37 exhibited comparatively high biofilm biomass at 37 °C despite moderate CSH and lower extracellular matrix-associated staining relative to its biomass measurement. This phenotype suggests that biofilm formation in J37 may involve additional surface or cellular determinants, although this possibility requires direct experimental investigation.

Hydrolytic enzyme activity was heterogeneous among the evaluated isolates. Esterase activity was widespread, whereas phospholipase activity varied among isolates and protease activity was uncommon. These enzymes are commonly considered virulence-associated phenotypes in *Candida* spp.; however, their detection in vitro should not be interpreted as direct evidence of pathogenicity. Similarly, the present study did not directly assess host-cell damage, tissue invasion, or in vivo virulence.

Zeta-potential measurements demonstrated substantial inter-isolate variation in cell-surface charge. CP1–CP4 exhibited more negative mean values, whereas CP6, CP7, and the marine isolates showed values closer to neutrality. Surface charge can influence microbial interactions with charged molecules and surfaces; however, the functional consequences of the observed variation were not directly assessed and require further investigation.

No detectable association was observed between fluconazole MIC variation and biofilm biomass at 25 °C under the tested conditions. Similarly, amphotericin B MIC values were not detectably associated with biofilm biomass at 25 °C, and phospholipase activity did not differ significantly between the two environmental sources examined. These analyses are restricted to the specific phenotypic outcomes measured and should not be interpreted as evidence for the presence or absence of an overall biological fitness cost.

From an ecological perspective, the recovery of *C. palmioleophila* from poultry litter and marine environments, together with previous reports from other environmental and animal-associated sources (Wang et al. 2021; Oliveira et al. 2025), demonstrates that this species occurs across diverse ecological settings. Nevertheless, the present sampling design does not allow these environments to be defined as epidemiological reservoirs, because environmental prevalence, persistence, and transmission were not evaluated.

From a One Health perspective, the recovery of *C. palmioleophila* from both agricultural and aquatic environments highlights the value of including environmental sources in surveillance of clinically relevant yeasts. However, the present study does not establish transmission between environmental, animal, and human compartments. Future studies combining systematic environmental sampling, clinical surveillance, and genomic epidemiology will be required to investigate population structure, environmental persistence, and potential transmission pathways.

Several limitations should be considered when interpreting these findings. First, the relatively small number of environmental isolates (*n* = 14) limits broad ecological comparisons between sources. Second, the study was designed for phenotypic characterization rather than estimation of environmental prevalence or demonstration of reservoir status. Environmental concentrations of antifungal compounds, agrochemicals, and other pollutants were not measured, preventing inference of local selective pressures. Third, ITS and D1/D2 sequencing were used for species identification rather than strain-level population analysis; therefore, genetic relatedness among isolates remains unknown. Finally, biofilm formation, CSH, hydrolytic enzyme activity, and thermotolerance represent phenotypes potentially relevant to pathogenicity but do not directly measure adhesion, host damage, tissue invasion, or in vivo virulence.

## Conclusion

In summary, this study demonstrates substantial phenotypic diversity among environmental *Candida palmioleophila* isolates recovered from poultry litter and urbanized coastal environments. The isolates exhibited high fluconazole MICs, growth under multiple stress conditions, thermotolerance up to 40 °C, biofilm formation, predominantly high cell-surface hydrophobicity, and variable hydrolytic enzyme activity, cell-wall chitin content, intracellular lipid-body accumulation, and cell-surface charge. Together, these findings expand current knowledge of the environmental phenotypic repertoire of this understudied yeast.

Importantly, the present study does not establish that the environments investigated select for antifungal resistance, constitute epidemiological reservoirs, or mediate transmission to humans or animals. Likewise, the phenotypes characterized here should not be interpreted as direct evidence of virulence or host adaptation. Broader environmental and clinical sampling, together with genomic characterization and functional studies, will be required to determine population structure, environmental persistence, potential selective pressures, pathogenic potential, and transmission pathways of *C. palmioleophila*. Within a One Health framework, these findings support the value of including environmental isolates in future investigations of the ecology and antifungal susceptibility of this emerging yeast.

## Data Availability

I will send the data by demand.
